# Digital screen time usage, prevalence of excessive digital screen time, and its association with mental health, sleep quality, and academic performance among Southern University students

**DOI:** 10.3389/fpsyt.2025.1535631

**Published:** 2025-03-24

**Authors:** Kamollada Kaewpradit, Pitchayanont Ngamchaliew, Napakkawat Buathong

**Affiliations:** Department of Family and Preventive Medicine, Faculty of Medicine, Prince of Songkla University, Hat Yai, Songkhla, Thailand

**Keywords:** digital screen time, excessive digital screen time, mental health, sleep quality, academic performance

## Abstract

**Background:**

Excessive digital screen time (EDST), which is defined as screen use that surpasses recommended limits, has been found to have detrimental effects on students’ mental health and academic performance. However, there is a paucity of studies investigating EDST in university students in Thailand.

**Objective:**

To investigate the prevalence, characteristics, and associations of excessive digital screen time with students’ mental health, sleep quality, and academic performance.

**Methods:**

A cross-sectional study was conducted at Southern University, Thailand, between December 2023 and January 2024. A total of 446 students completed self-administered questionnaires assessing DST characteristics, mental health, sleep quality, and academic performance. The tools used included the Depression Anxiety Stress Scale, Rosenberg Self-Esteem Scale, UCLA Loneliness Scale, and Pittsburgh Sleep Quality Index. DST was analyzed by device type and average weekly usage hours, focusing on smartphones, tablets, and computers. EDST was defined as daily usage exceeding 8 hours for smartphones, 6 hours for tablets, or 5 hours for computers. Participants exceeding these thresholds on any device were classified as having EDST. Sampling was conducted using quota sampling across faculties. Data were analyzed using chi-square tests, rank sum tests, and logistic regression, with significance set at P<0.05.

**Results:**

Students’ median age was 20 years (67.9% women). Most participants used smartphones for 4–6 hours daily (29.7%), tablets for <4 hours (29.8%), and computers for <4 hours (62.6%). Smartphones were primarily used for social media (73.1%), while tablets (28.4%) and computers (19.3%) were used for educational purposes. The prevalence of EDST was 48.4%, including 29.4% on tablets, 22.9% on smartphones, and 7.6% on computers. EDST was significantly associated with younger age (AOR 0.79; 95% CI 0.66–0.94) and enrollment in health science faculties (AOR 1.7; 95% CI 1.01–2.86).

**Conclusion:**

A high prevalence of EDST was observed among university students, particularly on smartphones and tablets. Younger students and those in health science programs were more prone to EDST, potentially due to higher academic demands and social media use. Interventions to enhance self-awareness, regulate screen time, and develop time management skills are recommended to mitigate its negative effects on mental health and academic performance.

## 1 Introduction

Digital screen time (DST), defined as the time spent in front of electronic device screens, has become a significant aspect of daily life. It is prevalent across all age groups in modern society, with smartphones, tablets, and computers occupying a significant portion compared to traditional devices such as television and video games (1). In 2023, the global average DST was 6 hours and 37 minutes per day, with mobile phones accounting for 3 hours and 46 minutes and computers for 2 hours and 51 minutes. Globally, Thailand ranked fourth in mobile phone use, averaging 5 hours and 5 minutes per day (2, 3). A survey revealed that approximately 95% of the Thai population uses smartphones, with the highest usage among individuals aged 15–24 at 99.2%; tablets were the next most utilized devices at 19.35% (4, 5). Additionally, a study by the Thai Ministry of Digital Economy and Society indicated that approximately 85% of Thai university students rely on smartphones as their primary digital device, underscoring the significant role of these devices in their daily routines (4). The younger generation exhibited particularly high DST, with individuals aged 22–41 averaging 8 hours and 55 minutes per day and those below 22 averaging 8 hours and 24 minutes (6). Activities include social media, communication, information searches, gaming, video streaming, online learning, and meetings (7).

Although DST offers benefits such as facilitating communication, education, and access to information (7), university students transitioning from adolescence to adulthood are particularly at risk due to high usage for both educational and leisure purposes (8). Excessive DST, defined as screen usage exceeding normal limits, has been associated with various psychological challenges, including depression, stress, anxiety, reduced self-esteem, and loneliness. Prolonged usage, particularly through social media, fosters unhealthy comparisons and contributes to increased stress (6, 8, 9), while constant connectivity and frequent notifications exacerbate anxiety (10, 11). A study conducted in the UAE identified significant correlations between depression and other adverse psychological conditions among university students. These conditions, including mood swings, insomnia, and addictive tendencies, were found to be directly linked to the duration of screen time on both weekdays and weekends (12). Feelings of exclusion or inadequacy during online interactions further diminish self-esteem and heighten loneliness (8, 13). Moreover, physiological effects such as compromised sleep quality—resulting from excessive screen exposure before bedtime—can lead to fatigue, mood disturbances, and poor academic performance (14–17). These sleep disruptions negatively affect overall well-being and academic outcomes, as poor sleep quality is associated with reduced cognitive function, shorter attention spans, and impaired memory retention (18, 19). In academic contexts, excessive DST has been shown to impair learning abilities, weaken critical thinking skills, and hinder academic performance (20, 21). The negative impacts on mental health, sleep, and academic success underscore the urgent need for intervention, particularly among university students.

Previous research has extensively examined DST and its effects, particularly in countries such as the United States (13), Saudi Arabia (8), the UAE (12), and China (6, 10, 14, 18). These studies highlight the importance of culturally specific investigations due to differences in educational systems, digital behaviors, and societal norms. However, research focusing on the Thai context remains limited despite Thailand’s unique digital landscape and high smartphone penetration. Existing studies in Thailand primarily address younger students or general usage patterns, leaving a significant gap in understanding the challenges faced by university students—particularly the effects of DST on mental health, sleep, and academic performance. This gap underscores the need for targeted investigations to provide insights into the specific experiences of Thai university students.

This study examines the characteristics and prevalence of DST and excessive DST among Thai university students, exploring their associations with mental health, sleep quality, and academic performance. The objective is to bridge gaps in existing knowledge and generate actionable insights for policymakers, educators, and health practitioners. Findings are expected to inform evidence-based interventions and institutional policies, fostering healthy screen time habits that enhance student well-being and academic outcomes.

## 2 Materials and methods

### 2.1 Study design and setting

A cross-sectional analytical study was conducted at the Prince of Songkla University (PSU), Hat Yai Campus, during the second semester of the academic year 2023, from December 1, 2023, to January 31, 2024. The study protocol was approved by the Office of Human Research Ethics Committee (HREC), Faculty of Medicine, PSU (REC.66-278-9-4). Informed consent was obtained from all participants before their enrollment in the study.

### 2.2 Participants and sample size calculation

Participants included 13,668 university students in the academic year 2023 at PSU on the Hat Yai Campus. The inclusion criteria were Thai undergraduate students aged 18 years and older who consented to participate. Individuals who self-reported a history of mental illness diagnosis or treatment were excluded from the study.

The sample size was calculated based on an assumed prevalence of 50% for depression, anxiety, stress, low self-esteem, loneliness, and poor sleep quality, as this assumption yields the largest sample size. Using a 95% confidence level and a 5% margin of error, the calculated minimum sample size was 375 participants. To account for potential exclusions due to incomplete data or non-responses, the target sample size was increased by approximately 20%, resulting in a final sample size of 450 individuals.

### 2.3 Data collection

Data were collected using paper-based questionnaires after business hours in public spaces such as cafeterias, libraries, and sports centers.

To minimize biases, quota sampling was employed to determine the number of participants required from each of the 16 faculties, ensuring proportional representation across academic disciplines. The allocated sample sizes were as follows: Faculty of Dentistry (n = 8, derived from 231 students), Faculty of Medical Technology (n = 8, derived from 223 students), Faculty of Nursing (n = 23, derived from 702 students), Faculty of Medicine (n = 43, derived from 1,319 students), Faculty of Pharmacy (n = 25, derived from 750 students), Faculty of Veterinary Medicine (n = 5, derived from 128 students), Faculty of Thai Traditional Medicine (n = 16, derived from 493 students), Faculty of Natural Resources (n = 27, derived from 811 students), Faculty of Science (n = 55, derived from 1,686 students), Faculty of Engineering (n = 75, derived from 2,292 students), Faculty of Agricultural Industry (n = 13, derived from 394 students), Faculty of Law (n = 35, derived from 1,068 students), Faculty of Management Sciences (n = 69, derived from 2,107 students), Faculty of Liberal Arts (n = 33, derived from 996 students), Faculty of Economics (n = 11, derived from 344 students), and the International College (n = 4, derived from 124 students). This resulted in a total sample size of 450 students.

Within each faculty, convenience sampling was used to recruit participants until the predefined quota was met. To prevent the overrepresentation of specific student groups, subsample sizes were capped proportionally to the total student population in each faculty. To further mitigate potential biases, unique identifiers were assigned to each participant, ensuring that each individual completed the questionnaire only once.

The faculties were categorized into three principal areas: Health Sciences (Faculties of Dentistry, Medical Technology, Nursing, Medicine, Pharmacy, Veterinary Medicine, and Thai Traditional Medicine); Science and Technology (Faculties of Natural Resources, Science, Engineering, and Agricultural Industry); and Social Sciences and Management (Faculties of Law, Management Sciences, Liberal Arts, Economics, and the International College). All questionnaires were completed anonymously, with ID numbers used solely for data analysis to ensure confidentiality. Access to the data was restricted to the research team to protect participants’ privacy.

### 2.4 Study tools

The questionnaire consisted of six sections: demographic data, DST usage, the Depression Anxiety Stress Scale (DASS-21), the Rosenberg Self-Esteem Scale-Revised (RSES-Revised), the Revised UCLA Loneliness Scale-Thai version, and the Thai version of the Pittsburgh Sleep Quality Index (T-PSQI).

Demographic data included age, sex, body mass index (BMI), relationship status, faculty, graduation year, and grade point average (GPA). Unsatisfactory academic performance was defined as a GPA below 3.00 in the latest semester.

Digital screen time (DST) is defined as the time spent in front of electronic device screens. DST usage was assessed by device type, average weekly usage hours, purpose of use, and duration before bedtime. The devices considered included smartphones, tablets, and computers. Participants reported their screen time in hours and minutes using their device’s operating system features: “Digital Well-being” on Android and “Screen Time” on iOS/iPadOS. The general prevalence of excessive DST was determined based on operational thresholds for each device type, derived from previous studies and expert consensus: more than 8 hours per day for smartphones (4, 8, 23, 24), more than 6 hours per day for tablets, and more than 5 hours per day for computers (10, 22, 25). Participants who exceeded these limits for one or more devices were classified as having excessive DST. This classification accounted for device-specific differences and provided a comprehensive assessment of overall DST behavior.

#### 2.4.1 The depression anxiety stress scale

The DASS-21 is a multidimensional mental health assessment tool with 21 questions that assess depression, anxiety, and stress. Developed by Lovibond (26) and adapted into Thai by Sawang et al. (27), it has Cronbach’s alphas of 0.82 (depression), 0.78 (anxiety), and 0.69 (stress). In this study, depression was defined as a score above 6 (moderate to extremely severe levels) (14); anxiety as a score above 7 (severe to extremely severe levels) (28); and stress as a score above 12 (severe to extremely severe levels) (28).

#### 2.4.2 The Rosenberg self-esteem scale-revised

The RSES-Revised, developed by Wongpakaran et al. (29, 30) based on the original RSES by Morris Rosenberg (31), consists of 10 questions with four response options each. It has a Cronbach’s alpha of 0.86 (30). In this study, scores <15 indicated low self-esteem, whereas scores ≥15 indicated high self-esteem (32).

#### 2.4.3 The revised UCLA loneliness scale-Thai version

Developed by Tinnakorn Wongpakaran and Nahathai Wongpakaran (33, 34) based on Russell’s UCLA Loneliness Scale Version 3 (35), this tool comprises 20 questions with reliability measured by Cronbach’s alpha, ranging from 0.80–0.88 (33, 34). Scores range from 20–80, with scores below 50 indicating low-to-moderate loneliness and scores of 50 or above indicating high loneliness (36–39).

#### 2.4.4 The Thai version of the Pittsburgh sleep quality index

Developed by Tawanchai Jirapramukpitak and Worawan Tanchaisawad (40) based on the original PSQI by Buysse et al. (41), the T-PSQI includes 9 questions that assess self-perceived sleep quality over the past month. The responses are categorized into seven components: subjective sleep quality, sleep latency, sleep duration, habitual sleep efficiency, sleep disturbances, use of sleep medications, and daytime dysfunction. The T-PSQI has a Cronbach’s alpha of 0.837 (42). Total scores range from 0–21, with scores ≤5 indicating good sleep quality and scores >5 indicating poor sleep quality.

### 2.5 Data analysis

Data from paper-based questionnaires were entered into Microsoft Excel to ensure accuracy. The R software version 4.3.3 was used for data cleaning and analysis. Descriptive statistics presented categorical variables as frequencies and percentages and continuous variables as medians and interquartile ranges (IQR). Categorical variables were analyzed using chi-square or rank-sum tests. Multivariate logistic regression models were used to explore the associations between excessive DST and the relevant variables. Variables with a p-value less than 0.2 in the univariate analysis, as well as those considered significant—including age, BMI, and faculty group—or meaningful to the outcomes—such as depression, anxiety, stress, low self-esteem, high loneliness, poor sleep quality, and unsatisfactory academic performance—were included in the multivariate analysis. Adjusted odds ratios (OR_adj_) and 95% confidence intervals (CI) were used to identify significant associations, with the significance level set at p < 0.05.

## 3 Results

**Figure 1:**
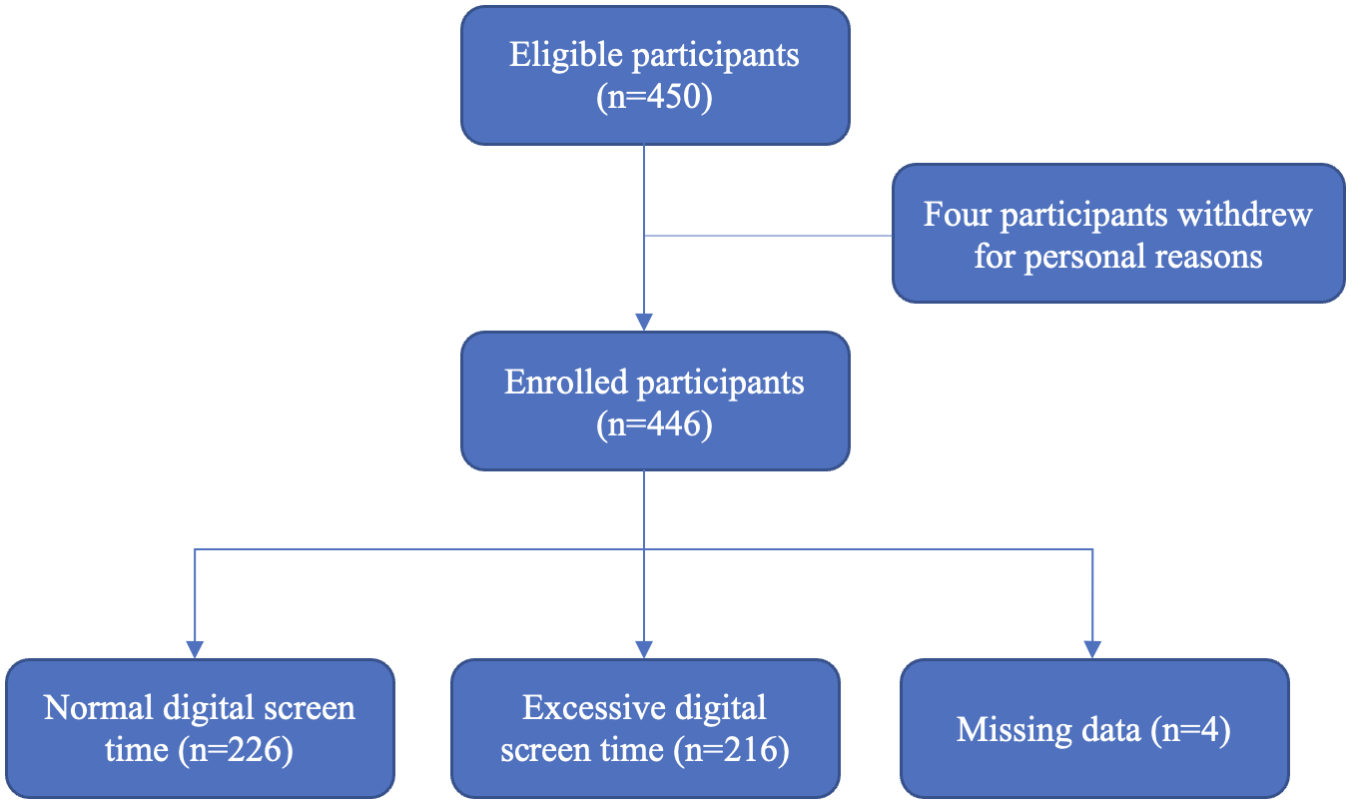
Flow diagram of study participants.

Of the 450 questionnaires distributed, 446 were completed, while four participants withdrew for personal reasons. An additional four participants had missing data in the DST section (**Figure 1**), leaving 442 participants for the analysis. The participants’ baseline characteristics are shown in **Table 1**. The median age was 21 years (IQR 18–26 years), and the majority were women (67.9%) and single (65.9%). Most participants were enrolled in Science and Technology programs (38.1%) and were in higher graduation years (4^th^ to 6^th^; 50.7%). The median GPA for the latest term and cumulative GPA were both 3.2 (IQR 0.92–4.00 and 1.68–3.98, respectively). Additionally, 31.3% of participants had unsatisfactory academic performance, defined as the latest GPA below 3.00.

**Table 1:** Baseline characteristics and divisions by excessive digital screen time (n=446).

Variable	Total (n=446)	Normal DST (n=226)	Excessive DST (n=216)	Crude OR (95% CI)	P-value
**Age (years)** **Median (IQR)**	21 (18,26)	21 (20,22)	21 (20,22)	0.81 (0.69,0.96)	0.046^a^
Sex, n (%)					0.745^b^
Male	143 (32.1)	70 (49.6)	71 (50.4)	Reference	
Female	303 (67.9)	156 (51.8)	145 (48.2)	0.92 (0.61,1.37)	
Body mass index (BMI), n (%)					0.19^b^
BMI < 25 **(non-obese)**	345 (77.5)	182 (53.1)	161 (46.9)	Reference	
BMI ≥ 25 **(obese)**	100 (22.5)	44 (44.9)	54 (55.1)	1.41 (0.89,2.25)	
Groups of faculties, n (%)					0.057 ^b^
Health Sciences	124 (27.8)	53 (42.7)	71 (57.3)	1.43 (0.88,2.35)	
Science and Technology	170 (38.1)	95 (56.9)	72 (43.1)	0.81 (0.52,1.28)	
Social Sciences and Management	152 (34.1)	78 (51.7)	73 (48.3)	Reference	
Graduation year, n (%)					0.509 ^b^
Lower graduation years (1^st^ to 3^rd^)	219 (49.3)	107 (49.3)	110 (50.7)	1.16 (0.79,1.68)	
Higher graduation years (4^th^ to 6^th^)	225 (50.7)	118 (52.9)	105 (47.1)	Reference	
Grade point average (GPA)					
Latest term GPA; median (IQR)	3.2 (0.92,4)	3.2 (2.8,3.5)	3.2 (2.9,3.5)	1.18 (0.8,1.74)	0.4^a^
Cumulative GPA; median (IQR)	3.2 (1.68,3.98)	3.2 (2.9,3.5)	3.2 (3,3.5)	1.25 (0.79,1.98)	0.392^a^
Status, n (%)					0.795^b^
Single	294 (65.9)	147 (50.5)	144 (49.5)	1.07 (0.73,1.59)	
Couple	152 (34.1)	79 (52.3)	72 (47.7)	Reference	
Mental health, sleep quality, and academic performance, n (%)					
Depression^p^	117 (26.2)	62 (27.4)	54 (25)	1.19 (0.77,1.84)	0.636 ^b^
Anxiety^q^	89 (20)	45 (19.9)	43 (19.9)	1.02 (0.64,1.65)	1 ^b^
Stress^r^	19 (4.3)	9 (4)	10 (4.7)	1.18 (0.47,2.96)	0.903 ^b^
Low self-esteem^s^	24 (5.4)	13 (5.8)	11 (5.1)	1.15 (0.48,2.72)	0.933 ^b^
High lonelinesst	83 (18.7)	42 (18.7)	40 (18.6)	0.97 (0.6,1.58)	1 ^b^
Poor sleep quality^u^	274 (61.9)	133 (59.1)	140 (65.1)	1.27 (0.86,1.88)	0.23 ^b^
Unsatisfactory academic performance^v^	135 (31.3)	71 (32.6)	62 (29.5)	1.18 (0.78,1.79)	0.565 ^b^

IQR, interquartile range; OR, odds ratio; CI, confidence interval; SD, standard deviation.

^a^ Rank sum test, ^b^ Chi-square test.

Health Sciences group: Dentistry, Medical Technology, Nursing, Medicine, Pharmacy, Veterinary Medicine, and Thai Traditional Medicine.

Science and Technology group: Natural Resources, Science, Engineering, Agricultural Industry.

Social Sciences and Management group: Law, Management Science, Liberal Arts, Economics, and the International College.

^p^Depression, DASS-21 subscale scores above 6; ^q^Anxiety, DASS-21 subscale scores above 7; ^r^Stress, DASS-21 subscale scores above 12; ^s^Low self-esteem, RSES-Revised scores below 15; ^t^High loneliness, Revised UCLA Loneliness Scale-Thai version scores above 49; ^u^Poor sleep quality, T-PSQI scores above 5; ^v^Unsatisfactory academic performance, latest semester GPA below 3.00.

Graduation year refers to the current year of study.

Status, n (%) refers to relationship status, indicating whether participants are single or in a relationship.

Characteristics of participants’ DST use are presented in **Table 2**. DST use varied by device; 97.3% of participants used smartphones, 68.4% used tablets, and 34.8% used computers. Most participants used smartphones for 4–6 hours per day (29.7%), tablets for <4 hours (29.8%), and computers for <4 hours (62.6%). Before bedtime, 59.2% of participants used smartphones for >30 min, whereas 54.1% and 73.5% used tablets and computers, respectively, for ≤30 min. The primary smartphone uses were social media (73.1%) and video streaming (17%). By contrast, tablets and computers were primarily used for educational purposes (28.4% and 19.3%, respectively) and video streaming (23.3% and 10.3%, respectively).

**Table 2:** Digital screen time use characteristics (n=442).

Digital screen time variable	Smartphone n = 434 (%)	Tablet n = 305 (%)	Computer n =155 (%)
Duration of daily DST (Average hours per week)			
4 hours or less a day	101 (23.3)	91 (29.8)	97 (62.6)
4 to 6 hours a day	129 (29.7)	83 (27.2)	33 (21.3)
6 to 8 hours a day	102 (23.5)	65 (21.3)	11 (7.1)
More than 8 hours a day	102 (23.5)	66 (21.6)	14 (9)
Duration of DST before bedtime			
≤30 minutes	177 (40.8)	165 (54.1)	114 (73.5)
>30 minutes	257 (59.2)	140 (45.9)	41 (26.5)
Purpose of DST use			
Access social media programs	326 (73.1)	51 (11.4)	6 (1.4)
Watch video/streaming	76 (17)	104 (23.3)	46 (10.3)
Use for education	8 (1.8)	127 (28.4)	86 (19.3)
Play games	8 (1.8)	14 (3)	22 (4.9)
Listen to music/podcasts	5 (1.1)	2 (0.4)	–
Online reading	4 (0.9)	1 (0.2)	–

The prevalence of excessive DST was 48.4%, with 216 of 446 participants exceeding the recommended usage limits. The percentages of excessive DST for each device are presented in **Table 3**. Specifically, 22.9% of the participants reported using smartphones for more than 8 hours per day, 29.4% used tablets for more than 6 hours, and 7.6% used computers for more than 5 hours. The prevalence of mental health conditions was assessed using standardized tools (**Table 1**). The results indicated that the prevalence of depression was 26.2%, while anxiety and stress were reported by 20% and 4.3% of the participants, respectively. Low self-esteem, high levels of loneliness, and poor sleep quality were observed in 5.4%, 18.7%, and 61.9% of participants, respectively.

**Table 3:** Percentage of excessive digital screen time for each device (n=442).

Type of device	Excessive digital screen time, n (%)
Tablet	131 (29.4)
Smartphone	102 (22.9)
Computer	34 (7.6)
All devices	216 (48.4)

Univariate analysis demonstrated significant associations between excessive DST and variables such as age, BMI, faculty group, and poor sleep quality (**Table 1**). Multivariate logistic regression further identified significant associations between excessive DST and age (adjusted OR 0.79; 95% CI 0.66–0.94) and the Health Science faculty group (adjusted OR 1.7; 95% CI 1.01–2.86, compared to the Social Sciences and Management group) (**Table 4**). Although factors such as mental health and academic performance were included in the analysis, no significant associations were observed between these variables and excessive DST.

**Table 4:** Multivariate logistic regression for factors associated with excessive digital screen time.

Variable	Crude OR (95% CI)	Adjusted OR (95% CI)	p-value (Wald’s test)	p-value (LR-test)
**Age**	0.81 (0.69,0.96)	0.79 (0.66,0.94)	0.009*	0.008*
Body mass index (BMI)				
BMI < 25 (non-obese)	Reference			
BMI ≥ 25 (obese)	1.41 (0.89,2.25)	1.5 (0.93,2.41)	0.095	0.094
Group of faculties				
Social Sciences and Management	Reference			0.019*
Health Sciences	1.43 (0.88,2.35)	1.7 (1.01,2.86)	0.0458*	
Science and Technology	0.81 (0.52,1.28)	0.86 (0.54,1.37)	0.522	
**Poor sleep quality**	1.27 (0.86,1.88)	1.37 (0.91,2.06)	0.129	0.128

Crude OR, crude odds ratio; Adjusted OR, adjusted odds ratio; CI, confidence interval.

Multivariate analysis using Wald’s test for regression analysis and the Likelihood Ratio test (LR test); * Statistically significant (p<0.05).

Poor sleep quality is defined as a Pittsburgh Sleep Quality Index (PSQI) score greater than 5.

## 4 Discussion

This study highlights the key aspects of DST among Thai university students. DST was categorized by average hours per week, revealing that most participants used smartphones for 4–6 hours per day, whereas tablets and computers were used for less than 4 hours. Smartphones were primarily used for social media, whereas tablets and computers were mainly used for educational purposes. Tablets also served as a replacement for physical books for over two-thirds of the participants. These findings support a survey by the Ministry of Digital Economy and Society (4) that reported that the average DST was highest for those aged 22–41 years (8 hours and 55 min) and <22 years (8 hours and 24 min). Similarly, a study by Maras et al. in Canada reported an average DST of 7–8 hours per day among a large community sample of adolescents (24). Additionally, research by Farzana Begum revealed that youth in Saudi Arabia spend an average of 7 hours daily on-screen media, with females averaging 8 hours and males 7 hours (8). Furthermore, a cohort study at the National University of Singapore found that first-year students spent an average of 14.3 hours daily on DST, with females reporting higher usage than males (23). These thresholds reflect the high prevalence of smartphone use and varying patterns of device usage. In line with previous research, DST was primarily dedicated to social media and entertainment, with educational use being secondary (13). Smartphones and tablets, with their advanced features, offer students a cost-effective alternative to computers, although computers remain crucial for certain educational tasks. While the NUS cohort study reported that daily DST was primarily for study purposes, averaging 7.6 hours per day, with computer usage being the highest at 7.0 hours per day (23), our study measured overall DST without differentiating between specific application usages. Participants reviewed their own device data to provide responses. Although inquiring about time spent on individual applications could help distinguish DST for academic versus recreational purposes, it might also raise privacy concerns and lead to underreporting, potentially resulting in an underestimation of actual DST usage.

This study found that almost half of the students experienced excessive DST, defined as >8 hours per day for smartphones, >6 hours for tablets, and >5 hours for computers. Currently, there are no standardized guidelines for appropriate DST limits. Although the definitions of excessive DST vary, previous studies consider 4–6 hours as high (10, 22, 25). While recommendations, such as those from the Canadian Society for Exercise Physiology, suggest limiting recreational screen time to no more than 3 hours per day for adults, the growing use of digital devices for various purposes beyond recreation necessitates further consideration (43, 44). Variability in DST criteria is influenced by differences in populations, age groups, countries, and cultures (45). This aligns with previous research. The high average DST observed in the Thai population (more than 8 hours) mirrors findings from a study by Maras et al., which reported an average DST of 7–8 hours per day among a large community of adolescents. That study, which examined the association between DST, depression, and anxiety, specifically noted cases where DST exceeded 8 hours per day. Additionally, the findings emphasized that prolonged total screen time was significantly correlated with more severe symptoms of anxiety (24). Meanwhile, in the United States, Madhav et al. (22) associated DST exceeding 6 hours with depression in adults. However, in China, Gao et al. linked DST exceeding 5 hours to high anxiety among college athletes (10).

This study further examined mental health aspects among university students, revealing a prevalence of 26.2% for depression, 20% for anxiety, and 4.3% for stress. Additionally, 5.4% of students reported low self-esteem, and 18.7% experienced high loneliness. In comparison, Chupradit et al. (46) found moderate to severe depression in 39.2% of Thai university students, severe anxiety in 25.8%, and high loneliness in 48.8%. Our study also found that 61.9% of participants had poor sleep quality, which is consistent with prior research (47).

The secondary aim of this study was to explore the association between excessive DST and various factors. The multivariate logistic regression analysis identified significant associations between excessive DST and both age and faculty groups. The adjusted odds ratio (AOR) of 0.79 (95% CI: 0.66–0.94) for age indicates that each additional year decreases the likelihood of excessive DST by approximately 21%. This decline can be attributed to cognitive and behavioral differences across age groups. Younger individuals are more likely to engage in screen-based activities, such as social media, gaming, and streaming, due to greater digital adaptability and heightened novelty-seeking tendencies.

This finding aligns with Matar Boumosleh et al. (48), who reported that younger individuals are more prone to excessive DST due to their reliance on digital platforms for academic, social, and entertainment purposes. Generational differences in technology use, along with a cultural emphasis on digital fluency, further reinforce this trend among younger students.

Regarding faculty groups, Health Science students were 1.7 times more likely to engage in excessive DST (AOR 1.7; 95% CI: 1.01–2.86) compared to Social Sciences and Management students. This association may be attributed to the rigorous academic demands of Health Science programs, which require prolonged use of digital devices for studying, accessing research articles, and participating in e-learning and simulation-based training. Additionally, the high -stress levels commonly associated with health-related studies may lead students to use digital platforms as a coping mechanism. These findings are consistent with Yeluri et al. (47), who observed elevated DST among medical students, particularly first -year students, due to their heavy reliance on electronic devices for academic purposes and the pressure of their demanding workload.

These results emphasize the significant influence of behavioral and environmental factors on excessive DST. Addressing these risks necessitates targeted interventions tailored to the specific needs of younger students and those in high-demand academic fields, such as Health Sciences, to promote healthier digital habits and mitigate potential negative consequences.

However, previous studies have shown that the impact of different types of DST on mental health varies (10, 22, 24, 49). For instance, smartphone and tablet use have been linked to mental health issues, whereas computer use has not demonstrated the same correlation. Ibrahim et al. (50) similarly found that excessive smartphone use in Saudi Arabia was associated with poor sleep quality and lower academic performance. Farzana (8) reported that over half of the youth in Saudi Arabia perceived DST as negatively affecting daily activities, sleep, academics, and family life, with strong associations between DST and feelings of depression (p=0.004) and loneliness (p=0.05).

Similarly, a four-year NUS cohort study revealed that high DST correlated with poor mental well-being (OR: 1.40; 95% CI: 0.99, 1.98) and psychological distress (OR: 1.56; 95% CI: 1.00, 2.44), particularly with recreational and study-related DST (OR: 1.81; 95% CI:1.27, 2.56; OR: 1.75; 95% CI: 1.11, 2.83) (23). Although studies have frequently reported a nonsignificant relationship between overall DST and sleep quality, DST use before bedtime may still influence sleep outcomes (47). In this study, factors such as depression, anxiety, stress, self-esteem, loneliness, sleep quality, and academic performance were analyzed; however, no significant associations with excessive DST were identified.

Despite these findings, addressing excessive DST in high-risk groups, such as younger students and those in health sciences programs, may still yield broader benefits by fostering healthier digital habits and supporting academic well-being. Practical recommendations include digital literacy programs to encourage responsible screen use and time management, offline study resources and structured device-free periods for health sciences students and low-tech learning options to promote a balanced approach to digital use. At the policy level, implementing mental health monitoring systems, creating screen-free zones, and launching awareness campaigns could further reduce excessive DST prevalence and enhance students’ overall well-being.

### 4.1 Strengths

The strength of this study lies in its rigorous methodological approach, particularly regarding data collection. First, the participants reported screen use using built-in system data or application records, which minimized recall bias by relying on actual usage data rather than memory. Second, to reduce the selection bias associated with online platform access, paper-based questionnaires were employed to ensure a balanced representation of the impact of DST. Third, data were collected during the second semester of the 2023 academic year, avoiding the examination period to minimize potential confounders such as exam-related stress and mental health challenges associated with adjusting to a new learning environment and meeting new peers. Finally, the increased use of online learning since the beginning of the COVID-19 pandemic may have led to results that differ from those of previous studies. The careful consideration of the timing and methodology strengthens the accuracy and reliability of the study’s findings.

### 4.2 Limitations and future directions

This study had certain limitations. First, the use of quota sampling followed by convenience sampling may introduce selection bias due to the overrepresentation of certain groups and unequal faculty representation. Additionally, non-response bias is possible, as participants may differ from non-participants in characteristics such as availability or interest in the study. The smaller sample size, which primarily consisted of female undergraduates, further limits the generalizability of the findings. Future studies should employ probability sampling and larger multicenter samples to enhance representativeness and robustness. Second, this study addressed potential biases in data collection from public spaces by assigning unique identifiers to participants to prevent duplicate responses. Recruitment efforts targeted students from diverse faculties and disciplines, and data were collected at various times and locations to improve representativeness. However, despite these efforts, the reliance on convenience sampling remains a limitation, which should be acknowledged when interpreting the findings. Third, DST was measured in total hours without differentiating its purpose (academic, work-related, or recreational). This limitation restricts a deeper understanding of how specific types of DST influence mental health. The findings reflect the overall impact of DST on mental health rather than the effects of distinct DST purposes. Future studies should categorize DST by purpose to clarify its unique effects on mental health, sleep quality, and academic performance. Fourth, although we referenced existing guidelines and research, relevant organizations have not provided definitive criteria for excessive DST. Finally, as this was a cross-sectional study, causality between excessive DST and mental health issues could not be established. Future studies should employ analytical methods to explore predictive factors and the causal relationship between excessive DST and mental health, as well as the impact of other variables on mental health.

## 5 Conclusion

Excessive DST was highly prevalent among Thai university students, with nearly half exceeding recommended usage limits, particularly on smartphones and tablets. This study identified significant associations between excessive DST and younger students, as well as those enrolled in health sciences programs, suggesting that these groups are at higher risk. Targeted interventions should prioritize these populations, implementing digital literacy programs to encourage balanced screen use and effective time management among younger students. For health sciences students, strategies such as integrating offline learning opportunities and establishing device-free study periods could help mitigate excessive screen exposure driven by academic demands.

## Data Availability

The datasets used and/or analyzed for the current study are available from the corresponding author upon reasonable request.
